# Basal Cell Cancer of the Scalp

**DOI:** 10.7759/cureus.26469

**Published:** 2022-06-30

**Authors:** Elizabeth O Amos-Arowoshegbe, Rio Varghese, Abia B Joseph, Chika Kanu-Ivi, Nehal Sadi, Sabina Sadana, Faisal Latif, Asiyah Abdul, Raunaq Ratra, Kyle Blume, Frederick Tiesenga

**Affiliations:** 1 Medicine, Windsor University School of Medicine, Basseterre, KNA; 2 Medicine, Saint James School of Medicine, West Virginia, USA; 3 Surgery, John F. Kennedy (JFK) University School of Medicine, Chicago, USA; 4 Surgery, Saint James School of Medicine, Park Ridge, USA; 5 Surgery, Saint James School of Medicine, Chicago, USA; 6 Surgery, Aureus University School of Medicine, Chicago, USA; 7 Surgery, Avalon University School of Medicine, Chicago, USA; 8 Surgery, Windsor University School of Medicine, Chicago, USA; 9 General Surgery, West Suburban Medical Center, Chicago, USA

**Keywords:** cosmetic dermatologic surgery, skin lesions, total excisional biopsy, skin cancer histology, nonmelanoma skin cancer, invasive scalp lesion, scalp lesion, skin cancer, uv light exposure, basal cell carcinoma

## Abstract

Basal cell carcinoma (BCC) is generally uncovered in sun-exposed areas, secondary to chronic unprotected UV exposure. The most common sites for nodular basal cells are the face, especially the nose, cheeks, forehead, nasolabial folds, and eyelids, with a history of crusting and friability. The commencement of BCC is 10 to 15 years from epidermal damage.

Here, we report the case of a 52-year-old Caucasian female who presented to her primary care with an enlarging bump on the scalp for the past five years, which became tender and friable two months before the visit. The patient was clinically diagnosed with a solitary cyst and was referred for surgical excision. The pathology of the excised specimen revealed it to be a BCC.

## Introduction

Basal cell carcinoma (BCC) is a common skin cancer with rare metastatic risk and is commonly found on the head and neck region due to protracted unprotected UV exposure [1]. Some studies have identified an increased incidence in women. The insidious onset of BCC is estimated to be 10-15 years from epidermal damage [2]. Another risk factor to consider is the time and length of sunlight exposure during childhood and adolescence [3]. Sunbathing, use of indoor tanning salons, history of sunburns, and a family history of BCC also contribute to the development of BCCs.

There are various morphological subtypes of BCC that exist, and they include nodular (solid), micronodular, superficial, cystic, infiltrating, infundibulocystic, pigmented, adenoid, metatypical, basosquamous, and fibro epitheliomatous tumors [4]. The most common is the nodular subtype, which typically presents as a shiny, pink- or flesh-colored papule or nodule with surface telangiectasia. These lesions may enlarge and ulcerate, giving the borders a rolled or rodent ulcer appearance. Superficial BCCs present as a pink-red, scaly macule or patch and contain telangiectasia and micro-ulcerations [4]. Clinically, superficial BCC can appear similar to inflammatory dermatoses such as eczema or psoriasis. Superficial basal cell carcinomas can evolve into nodular BCC over time [5].

## Case presentation

A 52-year-old Caucasian female patient referred by her primary care provider (PCP) came to the clinic with a chief complaint of an enlarging cyst on her scalp that was making her self-conscious because she could no longer cover it with her hair. Past medical history was notable for primary hypertension, depression, and migraines. Family history was significant for her father, who had been diagnosed with primary hypertension and leukemia, and a sister with primary hypertension. Surgical history was notable for hysterectomy roughly eight years ago.

The patient in this study is an immigrant to America from Poland who spent most of her youth working as a farmerette. While working on the farm, the patient identifies not being concerned about utilizing prophylactic protection for regular sun exposure. She is currently employed as a housekeeper in America, predominantly working weekdays while spending weekends engaged in various outdoor activities such as trips to the beach or skiing.

The patient inquired about the possibility of numerous trauma to the region as the probable cause of the enlarging cyst. There have been instances of trauma to the crown of her head. The most recent trauma occurred two weeks before coming to the clinic when she struck her head while exiting her car. This incident led to mild bleeding from the enlarging cyst.

The cyst first appeared four to five years ago as a small bump and slowly grew over the years. The patient started feeling mild-to-moderate pain with moderate friability in the area during the last two months. On physical examination, a solitary, round, non-tender cyst was seen on the anterior scalp. The skin surrounding the cyst was intact with mild crusting. There was no family history of such lesions. Vitals were stable.

**Figure 1:**
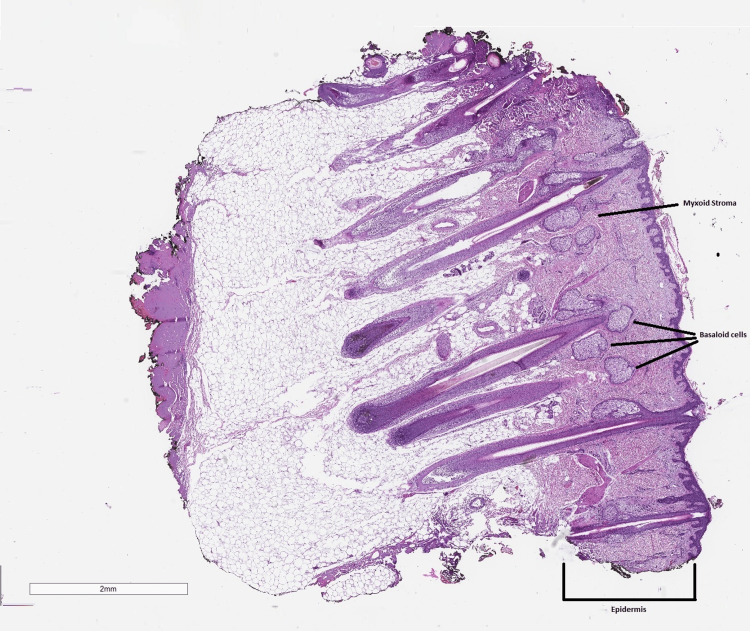
The basaloid cells that arise from the epidermis are most notable for diagnosing BCC. Careful analysis of the basaloid cells will show a peripheral palisading arrangement with peritumoral clefting (more prominent with higher magnification). Another useful distinguishing feature is the presence of myxoid stroma. BCC: Basal cell carcinoma.

With the tentative clinical diagnosis of a solitary cyst, incision and drainage of the cyst were planned. An elliptical incision of the mass down to the deep subcutaneous layer was made. A 2 cm x 3 cm mass was excised, and the specimen was sent to pathology. Per the pathology report, the specimen received in formalin labeled "scalp cyst" was gray/brown skin excision with underlying soft tissue measuring 2.4 cm x 1.3 cm x 1.0 cm. The epidermal surface is rough and consists of multiple hair follicles and one outgrowth measuring 10 mm x 8 mm. The interior is tan and homogenous upon sectioning. The specimen was confirmed as nodular BCC (Figure 1).

**Figure 2:**
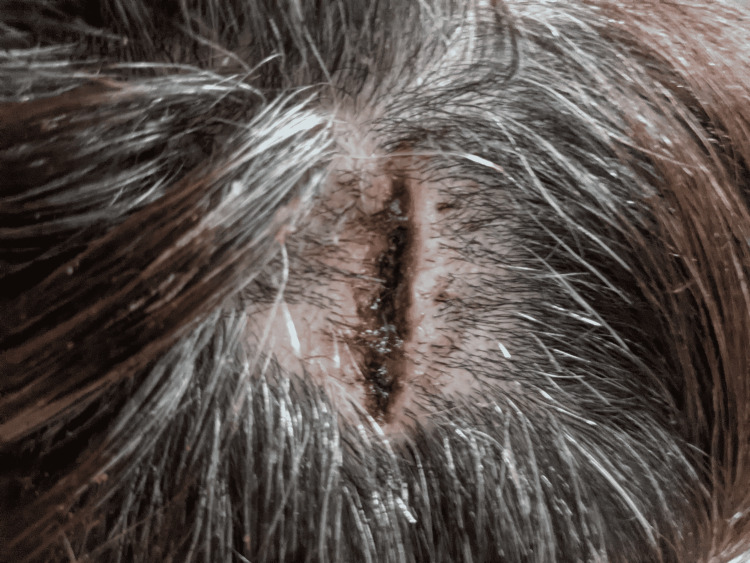
The scab on the site denotes the expected degree of healing for this time frame. Butadiene BID was placed to loosen up the scalp.

**Figure 3:**
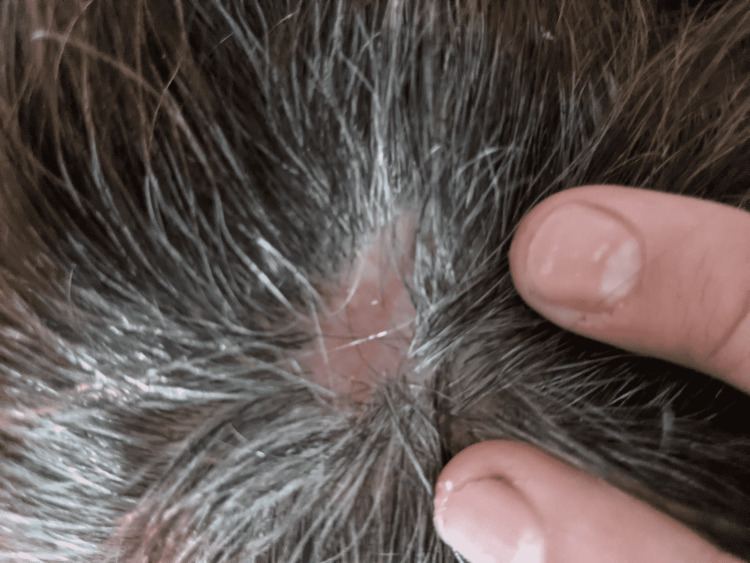
The incision site healed without residual scarring or marks. No new abnormalities were noted on examination.

**Figure 4:**
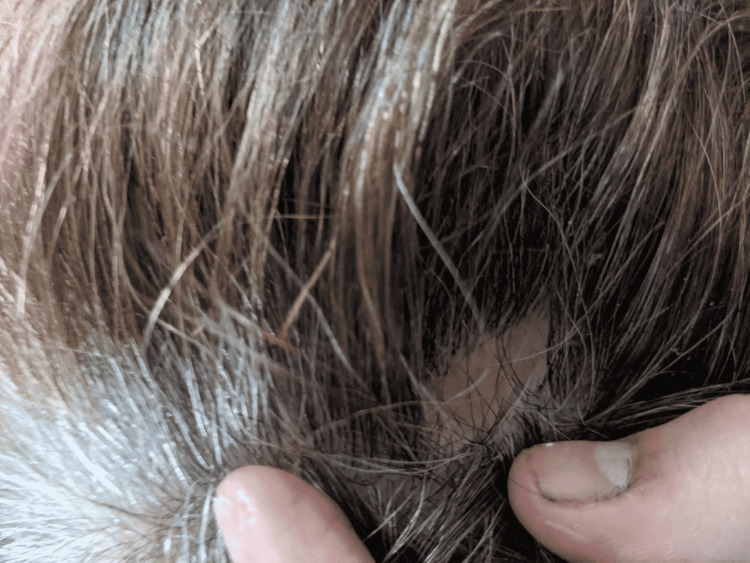
The hair at the incision site grew back. No noteworthy oddities were seen at the site.

After surgery, the patient was sent home with instructions to use butadiene BID to loosen up the scalp. On the follow-up visit on post-op day 8, the patient was doing well, and the incision site was healing properly with sutures coated with the scab (Figure 2). She denied pain, drainage, or fever. She followed up with oncology for final clearance and returned seven days later for the removal of sutures. In subsequent follow-up visits, the wound healed ably without prominent scarring or complications (Figure 3), with eventual regrowth of hair covering the region (Figure 4).

## Discussion

BCC formation can be caused by direct or indirect damage to DNA. Direct DNA damage occurs secondary to chronic UV light exposure, while indirect damages are due to reactive oxygen species and immune suppression. The patient lived in the farmlands of Poland during her childhood and worked as a farmerette before immigrating. According to a study from 2007 to 2013 on farmer groups in Poland, the leading risk factor of developing BCC is exposure to ultraviolet radiation (UVR) at a high dosage at a young age [6]. The patient reported that she enjoyed skiing and regularly visits the beach every summer, raising her exposure to UVR. 

The most often altered gene in BCC is the *PTCH1* gene that occurs in 70% of people with sporadic BCC [7]. About 10%-20% of people with sporadic BCC have smoothened (SMO) mutations. The second most common mutation found in BCCs is in the *P53 *gene. Mutations in the CDKN2A locus also have been detected in a smaller number of sporadic BCCs. Genetic contributions remain a possibility, but this patient was not genetically tested.

On histology, BCC presents as islands or nests of basaloid cells, with cells palisading at the periphery in a haphazard arrangement in the centers of the islands [8]. These small pleomorphic cells are composed of a basophilic nucleus without a discernible nucleolus and scanty cytoplasm [8]. Clefting is usually seen between the tumor and its surrounding stroma on paraffin-embedded sections. Mucin deposition is seen within the tumor and in the stroma around the tumor. The histologic differential diagnosis may include trichoepithelioma (rare benign lesion arising from hair follicles) or trichoblastoma (small benign hair follicle tumor originating from follicular germinative cells) [9].

Incisional, excisional, shave, and punch biopsies are generally used to diagnose BCC. In patients with obvious BCC and for whom the cosmetic outcome is not a priority, an excisional biopsy involving the removal of the entire lesion is done for histologic diagnosis and definitive therapy. Once confirmed through biopsy, treatment of BCC would depend on the location, type, and size. Mohs surgery is a micrographically controlled surgery under local anesthesia where the tissue is examined layer by layer. This method has the lowest recurrent rate for primary BCC. It has been reported that the five-year recurrence rate is 1.4% [10]. Other treatments include radiation therapy using high-energy beams such as x-rays and protons to treat any remaining remnant of the cancer cells. Electrodesiccation and curettage (E&D) are also commonly done, which involves surgical removal of the affected tissue with a curette with maximal margins. A side effect of E&D is the development of a hypopigmented scar. Topical treatment includes imiquimod 5% cream five times/week for six weeks, approved by the FDA for treatment of superficial BCC in low-risk zones, resulting in a histologic clearance rate of 82% 12 weeks after the treatment [11].

The tentative diagnosis was a solitary cyst prior to the E&D of this lesion in this report. The patient desired the removal of the lesion due to increasing pain, discomfort, and mild friability. The belief that the lesion was a solitary cyst remained due to its location until pathology confirmed it as a nodular BCC. Nodular BCC remains a rare finding on the scalp.

## Conclusions

Among skin cancers, BCC is common. Typically present as a shiny, pink papule or nodule with surface telangiectasia. Lesions can often enlarge over time or bleed. Most commonly found on the face, the disease usually develops due to chronic exposure to UV radiation, causing direct DNA damage. Due to its highly variable presentation and insidious onset, diagnosing BCC comes with its challenges. Its ability to disguise as other more benign lesions makes it easy to misdiagnose. Patients can also delay diagnosis, such as in this case, especially when there are no alarm symptoms, such as bleeding or itching in the area, leading to continued growth without interruption. The primary care physician was acquainted with the lesion after it became friable because the patient became alarmed.

This case adds to the discussion of BCC as there are many unique features. The location was noteworthy as the most common sites for nodular basal cells are the face, especially the nose, cheeks, forehead, nasolabial folds, and eyelids. In this patient, BCC was found on the scalp with moderate friability. The time of onset and the growth rate was also significant as BCC in this patient presented itself as a slow-growing cyst with a prolonged growth rate, initially presenting itself approximately five years ago. This case highlights the importance of using protective gear such as hats, especially with chronic exposure to sunlight. Patients should be educated concerning seeking medical care even in the absence of alarming symptoms, preventing delays in diagnosis and intervention. 
